# Retroperitoneal Hematoma Caused by Lumbar Artery Injury during Percutaneous Disc Nucleoplasty: Successful Treatment Using Emergent Transarterial Embolization

**DOI:** 10.3390/diagnostics12071568

**Published:** 2022-06-28

**Authors:** Dae Kyun Kim, Seok Won Kim

**Affiliations:** Department of Neurosurgery, College of Medicine, Chosun University, Gwangju 61453, Korea; dae2842@naver.com

**Keywords:** disc herniation, arteries, hematoma

## Abstract

Although minimally invasive percutaneous disc nucleoplasty (PDN) has been widely used for discogenic back pain or soft-disc herniation of the lumbar spine, complications can occur. Although rare, iatrogenic vascular injuries during PDN are serious complications that can be fatal without rapid diagnosis and effective management. Here, we present the case of a 38-year-old male patient with an injury to the right fifth lumbar artery that occurred during PDN, which was successfully treated using emergent transarterial embolization. To the best of our knowledge, this is the first report of successful transarterial embolization without open hematoma evacuation for a lumbar artery injury that occurred during PDN.

**Figure 1:**
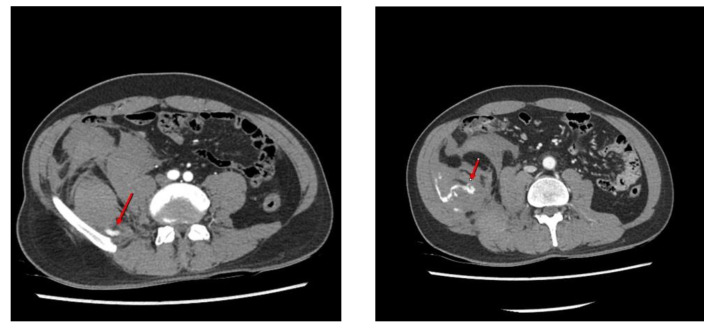
Although microscopic discectomy is still the mainstay of surgical treatment for symptomatic lumbar disc herniation, percutaneous disc nucleoplasty (PDN) is a new minimally invasive procedure for lumbar disc herniation. Given the current trends in minimal access surgery, PDN is considered a relatively safe and effective procedure and has been widely performed by spine surgeons [1,2]. A 38-year-old man presented with radiating pain in the left buttock and leg (S1 dermatomes) that started several months before visiting our outpatient clinic. Lumbar magnetic resonance (MR) imaging revealed central and left lateral-recess disc protrusion at the L5-S1 level. Percutaneous disc decompression using coblation technology (nucleoplasty) was performed in the angiography room, with the patient in the prone position under local anesthesia. A 17-gauge introducer cannula was introduced, using the posterolateral approach, into the identified disc level and advanced into the symptomatic portion of the disc under fluoroscopic guidance. The introducer needle was inserted into the L5-S1 disc, during which a failure to insert the needle resulted in several punctures of the lateral bone of L5. After the introducer needle insertion, the surgical probe was placed into the introducer needle and advanced until the probe tip contacted the annulus on the symptomatic side. Ablation was performed for 20–60 s and retreated four times to coagulate the intervertebral disc (L-disq^®^, U&I company, Uijeonbu, Korea). After the procedure, he reported a slight improvement in his left lower extremity symptoms. However, he complained of newly developed abdominal pain in the right flank region in the recovery room. Physical examination revealed tenderness and swelling in the right abdomen. Contrast-enhanced computed tomography (CT) of the abdomen and pelvis revealed an acute right retroperitoneal hematoma due to active bleeding caused by an L5 lumbar artery injury (Figure 1). Contrast-enhanced computed tomography images show high attenuation (arrows) from the right fifth lumbar artery within the hematoma, indicating active bleeding.

**Figure 2:**
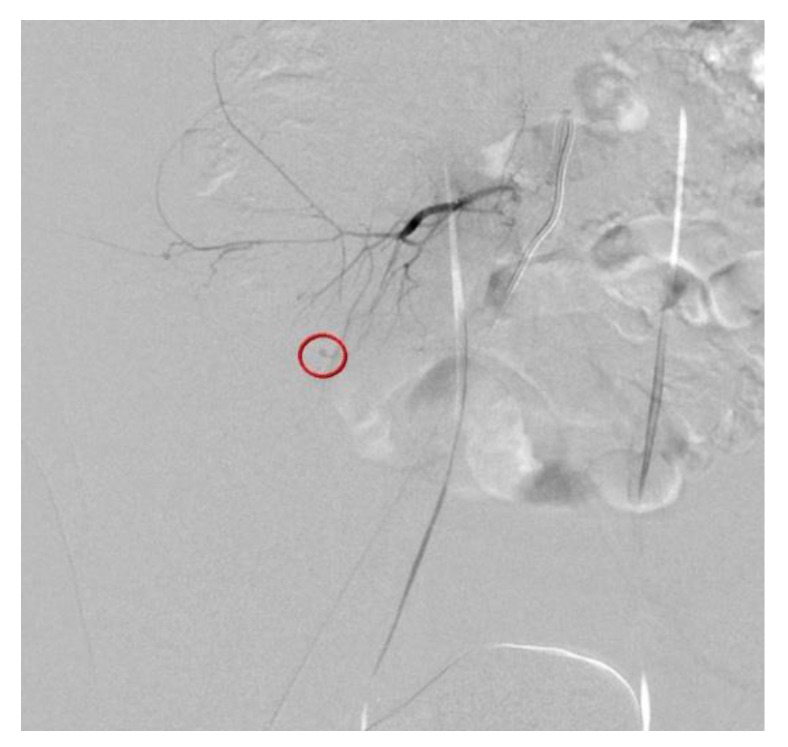
Due to constant abdominal tenderness, flank pain, and tachycardia, selective angiography was performed. The contrast medium was extravasated from the distal fine branch of the right fifth lumbar artery (Figure 2). Selective angiography shows that the contrast medium is extravasated (circle) from the distal fine branch of the right fifth lumbar artery.

**Figure 3:**
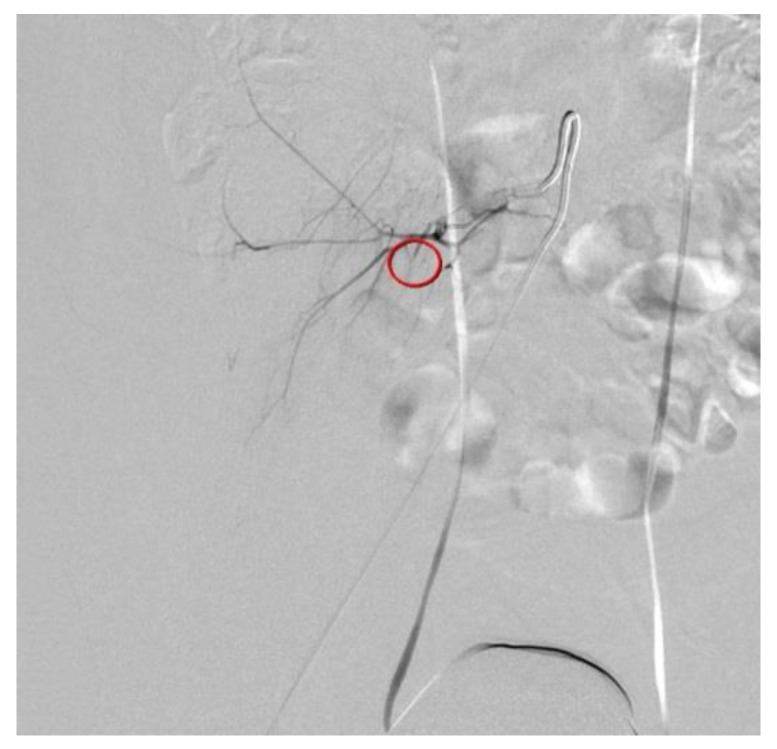
Transcatheter arterial embolization (TAE) of the ruptured fifth lumbar artery was successfully performed using Gelfoam particles (Figure 3). The patient recovered uneventfully and was discharged 4 days after TAE, without any complications or blood transfusion. After 1 month of follow-up, the patient recovered uneventfully, and no hemorrhage-related complications were observed. Iatrogenic lumbar artery injury during PDN is rare, but may be a serious condition that requires early detection and urgent treatment. Great care should be taken to avoid hemorrhagic complications, and adequate an technique and anatomical considerations are important to avoid these complications. Transarterial embolization, rather than open hematoma evacuation or laparotomy, can be a safe and effective treatment to stop active bleeding. Successful embolization of the bleeding vessel is performed using Gelfoam particles (circle).

## Data Availability

Not applicable.
